# A Molecular Perspective on HIF-1α and Angiogenic Stimulator Networks and Their Role in Solid Tumors: An Update

**DOI:** 10.3390/ijms25063313

**Published:** 2024-03-14

**Authors:** Anuja Gajanan Magar, Vivek Kumar Morya, Mi Kyung Kwak, Ji Ung Oh, Kyu Cheol Noh

**Affiliations:** 1 Hallym University Dongtan Sacred Heart Hospital, Dongtan 18450, Republic of Korea; 2 School of Medicine, Hallym University, Chuncheon-si 24252, Republic of Korea

**Keywords:** hypoxia, HIF-1α, solid tumors, angiogenesis, VEGF, angiopoietins

## Abstract

Hypoxia-inducible factor-1α (HIF-1α) is a major transcriptional factor, which plays an important role in cellular reprogramming processes under hypoxic conditions, which facilitate solid tumors’ progression. HIF-1α is directly involved in the regulation of the angiogenesis, metabolic reprogramming, and extracellular matrix remodeling of the tumor microenvironment. Therefore, an in-depth study on the role of HIF-1α in solid tumor malignancies is required to develop novel anti-cancer therapeutics. HIF-1α also plays a critical role in regulating growth factors, such as the vascular endothelial growth factor, fibroblast growth factor, and platelet-derived growth factor, in a network manner. Additionally, it plays a significant role in tumor progression and chemotherapy resistance by regulating a variety of angiogenic factors, including angiopoietin 1 and angiopoietin 2, matrix metalloproteinase, and erythropoietin, along with energy pathways. Therefore, this review attempts to provide comprehensive insight into the role of HIF-1α in the energy and angiogenesis pathways of solid tumors.

## 1. Introduction

Cancer is considered a major concern in this aging world. The World Cancer Statistics Report (2020) reported about 10 million mortalities from 19.3 million new cancer cases (excluding non-melanoma skin cancer) [1]. The Cancer Statistics Report (2018) revealed that Asia accounts for more than half of the world’s cancer incidence, followed by Europe with 23.4% and America with 21% [2]. In 2023, the American Cancer Society reported 1,958,310 new cases and 609,820 deaths in the United States [3,4]. It is estimated that by 2040, there will be 28–29.4 million cases (approx.) per year (worldwide) [1,5]. Among all types of cancers, approximately 85% of cancer-related deaths are due to solid tumors [6,7]. Solid tumors induce various genetic modifications, such as the activation of angiogenic factors, elevated glycolysis, and the repression of tumor suppressor genes, which contribute to the immunosuppressive tumor microenvironment (TME) [8,9,10,11,12]. This allows tumors to propagate and develop a resistance against immune cells, immunotherapies, and radiotherapies [1,8,13]. Hypoxia plays a crucial role in the TME, which helps tumor formation and progression [10,11,12].

The hypoxia-inducible factor (HIF), a primary regulator under hypoxic conditions, has many isoforms, such as HIF-1α, HIF-2α, and HIF-3α. However, HIF-1α and HIF-2α are primarily expressed under hypoxic conditions. HIF-2α, a structural homolog of HIF-1α, exhibits a diverse nomenclature, including endothelial PAS protein-1, HIF-related factors, and HIF-like factors. Despite sharing similar structural features with HIF-1α, HIF-2α demonstrates distinct tissue-specific expression patterns [14]. HIF-1α primarily regulates the expression of genes associated with angiogenesis, proliferation, cell migration, and glycolysis in solid tumor cells [9,10,11]. Therefore, it is considered a key cytological regulator of cancer cell growth and survival [11]. The expression of HIF-1α and HIF-2α are different; unlike HIF-1α, HIF-2α is mainly reported in the lung, carotid body, and endothelium [15]. HIF-2α is mainly involved in the fatty acid metabolism, whereas HIF-1α controls glycolysis in solid tumors; however, in some pathways, their roles overlap [16]. HIF-2α is involved in regulating cell reprogramming and pluripotency by targeting Oct4 and transforming growth factor-α (TGF-α) [15]. However, the third isoform, HIF-3α, acts as a dominant-negative regulator of HIF-1α and HIF-2α, and its expression is limited to the eye and cerebellum [17]. However, the primary function of this isoform remains unclear. Both HIF-1α and HIF-2α contribute to tumor hypoxia, but HIF-1α appears to be more prominently involved in energy metabolism and angiogenesis [18,19]. Many studies have suggested that HIF-1α is a key regulator of many essential pathways, including those of growth factors, glycolytic enzymes, the modulation of natural killer cells, resistance to chemotherapy and radiotherapy, and the promotion of angiogenesis. [20,21,22]. Recent studies revealed that HIF-1α-dependent genes play a crucial role in the rapid adaptation of tumor cells under hypoxic conditions by reprogramming the cellular metabolism [17,23]. Therefore, the present article focuses on the role of the HIF-1α and angiogenic factor network, and metabolic alterations in the solid TME.

Several studies have suggested that HIF-1α is the major transcriptional regulator of solid tumors [9,10,11]. HIF-1α expression regulates the transcription of angiogenic factors, such as the vascular endothelial growth factor (VEGF), platelet-derived growth factor (PDGF), TGF, angiopoietins (ANG), erythropoietin (EPO), and fibroblast growth factors (FGF) [6,12,13,24]. HIF-1α also stimulates proteolytic enzymes such as matrix metalloproteinase (MMP), collagenase, and fibronectin to remodel the extracellular matrix (ECM) for tumor survival [25,26]. This helps in angiogenesis via the migration of tumor-adjoining endothelial cells to develop new blood vessels [25,27]. These new vesicles are helpful for tumor survival, as they provide oxygen and fundamental metabolic requirements to solid TME [27,28,29]. This leads to metabolic reprogramming, which triggers the Warburg effect. The Warburg effect creates a pseudo-hypoxic environment that induces HIF-1α expression [30]. The expression of HIF-1α is elevated and stabilized by the lactate (a byproduct) produced during the Warburg effect [31]. This results in the switch from oxidative phosphorylation (OXPHOS) to accelerated glycolysis in tumor cells, which aids in cancer cell adaptation to hypoxic environments [32]. Due to the hypoxic environment, an aggressive tumor phenotype is observed, which shows resistance to chemotherapy, radiation therapy, and piteous responses to anti-tumor therapy [33]. The treatment of solid tumors remains a Herculean task owing to the involvement of numerous variables involved in tumor growth, metastasis, and tumor survival [7,34]. HIF-1α is a critical factor in these processes, as it is involved in various essential signaling cascades, and each of them could be a primary target for developing therapeutics [35]. Therefore, HIF-1α is a key contributor to non-significant treatment results, disease progression, and recurrence in solid tumors [9,10]. It is also a key regulator of the upstream and downstream metabolic processes of cancer cells, which are required for the emergence of the invasive, metastatic, and fatal cancer phenotype [36].

The high recurrence rate of solid tumors requires research focusing on novel therapeutic strategies that target essential proteins. Although numerous cancer treatments exist, their efficacy remains limited [37]. HIF-1α, a transcription factor that activates multiple pro-tumorigenic signaling pathways, could be a better target in association with the angiogenic regulator network. Therefore, a detailed understanding of HIF-1α and its signaling cascade is crucial for identifying specific targets and pathways that can be effectively inhibited to halt solid tumor development.

## 2. HIF 

Tumor hypoxia, defined as oxygen deprivation within tumor tissues, was first recognized as a pivotal factor in cancer progression in the 1950s [38]. Subsequent research has confirmed its status as a potent risk factor for poor patient outcomes [38]. The HIF family of transcriptional regulators is a key mediator of cellular adaptation to hypoxic stress [39]. These heterodimeric complexes comprise an oxygen-sensitive α subunit (HIF-α) and a constitutively expressed β subunit (HIF-β) [40,41,42,43]. To date, three isoforms of HIF-α have been identified: HIF-1α, HIF-2α, and HIF-3α [39,40]. Both HIF-1α and HIF-2α have been extensively linked to oncogenic gene expression and the aggressive behavior of solid tumors [10,35]. Conversely, the functional role of HIF-3α remains less understood, with limited research currently available [31,41].

### HIF-α and HIF-β Subunits: Regulatory Players in Cellular Oxygen Response

The HIF-α and HIF-β subunits belong to the basic helix-loop-helix-Per ARNT SIM (bHLH-PAS) protein family and are characterized by their modular structure and roles in oxygen-dependent transcriptional regulation [44]. Although HIF-α subunits exhibit substantial diversity (e.g., HIF-1α and HIF-2α share only 48% overall amino acid similarity), they possess highly conserved bHLH domains (83% similarity), facilitating heterodimerization with the constitutively expressed HIF-β subunit also known as the aryl hydrocarbon receptor nuclear transporter (ARNT) [42,43,44]. Interestingly, both HIF-1α and HIF-2α undergo similar oxygen-dependent hydroxylation events, highlighting the shared regulatory mechanisms governing their stability and activity [42,43,44]. ARNT functions as a versatile adaptor protein, interacting not only with HIF-α subunits but also with the aryl hydrocarbon receptor. This interaction is critical for shuttling the HIF-α/HIF-β complex into the nucleus under hypoxic conditions, enabling the subsequent transcriptional activation of target genes [44]. More than 100 genes are regulated by HIF-1α, which promotes cellular adaptation and survival in low-oxygen environments [11]. This adaptive response plays a crucial role in various physiological and pathological processes, including tumorigenesis.

**Figure 1:**
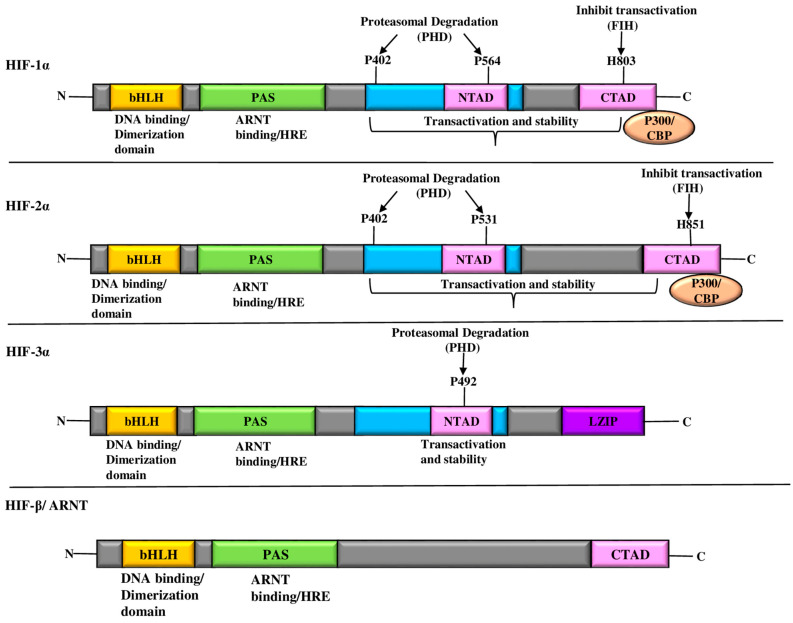
Diagrammatic representation of the domain structures of HIF. The HIF-1α, HIF-2α, and HIF-3α structural domains along with their transcriptional binding partner, HIF-1β/ARNT, comprise the (HIF-α/HIF-1β) transcriptional complexes. The NH2-terminal region of HIF-α and HIF-1β contains bHLH and PAS domains, which are essential for heterodimerization and DNA binding in the HRE at the target gene loci. HIF-α has two TADs at its COOH-terminus. The ODD domain of HIF-1α, HIF-2α, and HIF-3α contains proline(s) residues. HIF-3 has a LZIP domain in the COOH-terminal region; it also lacks the TAD-C domain, unlike HIF-1α, HIF-2α. Abbreviations: ARNT: aryl hydrocarbon receptor nuclear translocator; bHLS-PAS: basic helix loop helix-per ARNT sim; CBP: CREB-binding protein; FIH: factor-inhibiting hypoxia-inducible factor; HIF: hypoxia-inducible factor; HRE: hypoxia-responsive element; LZIP: leucine zipper; PHD: prolyl hydroxylase domain; TAD: trans-activation domain.

HIF-α subunits contain a distinctive domain structure composed of N-terminal, C-terminal, and PAS domains (Figure 1). The N-terminal bHLH domain is involved in DNA-binding and heterodimerization with HIF-β to form the core functional unit. The central PAS domain, known for its protein–protein interaction and oxygen-sensing capabilities, contributes to regulatory functions [45,46]. The C-terminal domain contains two transactivation domains (TADs), designated N-TAD and C-TAD [45,46]. Proline residues P402 and P564 of the N-TAD are crucial for protein stability under hypoxic conditions [47]. In contrast, the C-TAD contains the arginine residue N803, which is responsible for modulating HIF-1α transcriptional activity through post-translational modifications [47]. The dimerization interface of HIF-β subunits adjacent to the N-terminal bHLH domain underscores its structural importance in complex formation [11,38].

## 3. HIF-1α-Mediated Metabolic Reprogramming in Hypoxia and its Role in Tumorigenesis

Hypoxia, defined as limited oxygen availability, poses a significant challenge to cellular homeostasis. Initially, it triggers a compensatory mechanism to increase oxygen delivery; however, prolonged hypoxia can lead to cellular dysfunction and proliferation in an attempt to overcome hypoxic conditions [44,48]. This demand creates an imbalance between the oxygen supply and utilization, which is helpful for tumor formation [44]. For tumor growth and proliferation, cancer cells undergo metabolic reprogramming involving alterations in glycolysis, mitochondrial biogenesis, the lipid and amino acid metabolism, and the pentose phosphate pathway (PPP) [44]. Studies suggest that HIF-1α plays an important role in orchestrating these metabolic adaptations, thus promoting cell survival and developing an immunosuppressive TME, which is helpful for malignant progression [48].

### 3.1. Warburg Effect: OXPHOS to Accelerated Glycolysis

**Figure 2:**
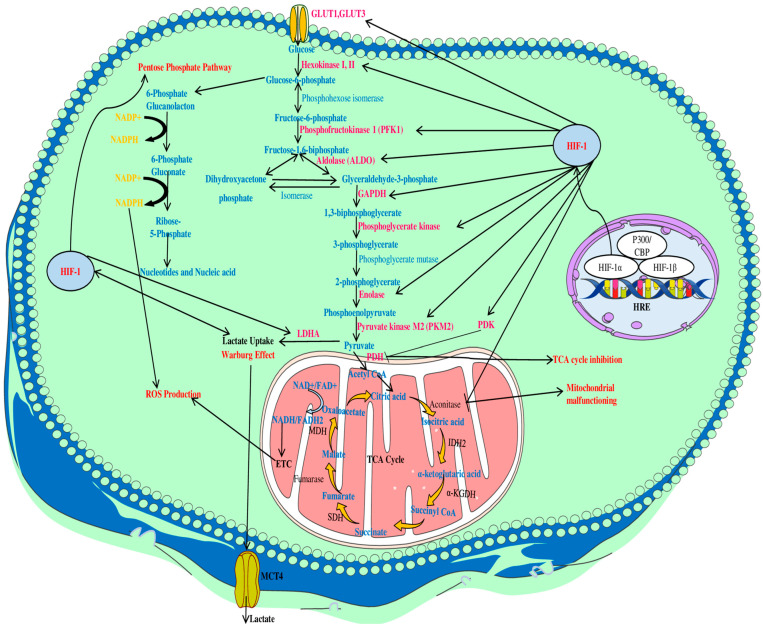
Significance of the HIF-1α-signaling pathway in metabolic reprogramming in solid tumors. In hypoxic scenarios, HIF-1α enhances glucose uptake by positively regulating glycolytic enzymes and inducing the expression of PDK, the negative regulator of PDH, which results in the conversion of pyruvate into lactate instead of acetyl-CoA. This reduction of acetyl-CoA results in the inhibition of the TCA cycle; however, it also causes mitochondrial malfunctioning by inhibiting the activity of aconitase. HIF-1α also produces higher levels of ROS in cancer cells through the inhibition of the TCA cycle and by activating the pentose phosphate pathway. Abbreviations: Acetyl CoA: acetyl coenzyme A; CBP: CREB-binding protein; ETC: electron transport chain; FADH2: flavin adenine dinucleotide; GLUT: glucose transporters; GAPDH: glyceraldehyde-3-phosphate dehydrogenase; HIF-1: hypoxia-inducible factor-1; HRE: hypoxia-responsive element; IDH2: isocitrate dehydrogenase 2; KGDH: ketoglutarate dehydrogenase; LDHA: lactate dehydrogenase A; MDH: malate dehydrogenase; MCT4: mono-carboxylate transporter4; NADP: nicotinamide adenine dinucleotide phosphate; NADPH: nicotinamide adenine dinucleotide phosphate hydrogen; PDH: pyruvate dehydrogenase; PDK: pyruvate dehydrogenase kinase; ROS: reactive oxygen species; SDH: succinate dehydrogenase; TCA cycle: tricarboxylic acid cycle.

In normal cells, the primary means of generating the energy molecule adenosine 5′-triphosphate (ATP) is through mitochondrial OXPHOS in the presence of oxygen [32,48]. The glycolysis of glucose in the cytoplasm and the citric acid cycle in the mitochondria are well-known processes by which normal cells oxidize glucose (Figure 2). During glycolysis, glucose undergoes oxidation and reduction reactions, resulting in the production of two pyruvate molecules as end products. These pyruvates are then transported to the mitochondria, where they oxidize to form acetyl coenzyme A (acetyl-CoA) [49]. This acetyl-CoA is a crucial precursor of the tricarboxylic acid cycle (TCA cycle), which generates electron donors from nicotinamide adenine dinucleotide (NADH) and flavin adenine dinucleotide (FADH2). Additionally, these newly formed electron donors move towards the electron transport chain (ETC) in mitochondria and combine with the electron acceptor O_2_ [31]. The oxidation of these energy molecules through OXPHOS results in the generation of 36 ATP molecules, which serve as fuels for cellular functions [49]. Healthy cells adapt to the greater activity of OXPHOS to provide energy for cell development, proliferation, and other functions [31].

Oxygen is essential for both normal and malignant cells as it enables the production of substantial quantities of energy in the presence of dextrose. Normal cells cease to proliferate in the absence of oxygen, whereas tumor cells continue to proliferate. Beyond critical tumor mass growth, existing blood vessels are unable to supply oxygen and nutrients to tumor cells [49]. However, cancer cells’ survival and proliferation under hypoxic conditions remain unclear and are an ongoing area of research. Warburg et al. reported that cancer cells exhibit a high rate of dextrose uptake, perform the Embden–Meyerhof pathway, and enhance lactate generation [49,50]. Tumor cells prefer an accelerated Embden–Meyerhof pathway followed by lactate fermentation over OXPHOS, even under normal oxygen conditions, a phenomenon known as the “Warburg effect” [32,49,50]. This process is crucial for cancer cells; it also allows the intake of additional metabolites by the cells, such as nucleoside triphosphate, thereby promoting enhanced cellular proliferation and growth (Figure 2) [44].

### 3.2. Effect of HIF-1α on the Activation of the Pentose Phosphate Pathway

The energy metabolism is significantly reprogrammed in solid tumors, which promotes the de novo biosynthesis of essential metabolites, alterative ATP generation, and detoxification. These collective advantages promote the rapid proliferation and growth of tumor cells [51]. Among these, the PPP has been identified as the major pathway for glucose catabolism in tumor cells under hypoxic conditions [48]. Under hypoxic conditions, ribose 5-phosphate produces several intermediate products, which are required as precursors for nucleotide biosynthesis. HIF1-α plays an important role in this metabolic alteration along with other oncogenes including Myc and phosphatidylinositol 3-kinase/protein kinase B (PI3K/Akt) [52]. Hypoxia also plays an important role in the inactivation of tumor suppressors such as p53 [53]. Naturally, tumor suppressor protein 53 (p53) exerts regulatory control over both oxidative and non-oxidative PPP and promotes apoptosis. However, under hypoxic conditions, the overexpression of HIF-1α reduces p53 protein levels and attenuates p53 transcriptional activities by competing for p300, a coactivator required for the full activities of both p53 and HIF-1α [53]. The p53-induced glycolysis and apoptosis regulator, a direct downstream target of p53, suppresses phosphofructokinase-1 expression, thereby modulating glycolysis and channeling substrates towards the TCA cycle [54]. However, most cancer cells have mutations or deletions that inactivate p53, leading to dysregulated PPP activation. Glyceraldehyde 3-phosphate dehydrogenase also contributes to PPP regulation. Conversely, OXPHOS suppression downregulates glyceraldehyde 3-phosphate dehydrogenase, leading to the accumulation of glycolytic intermediates that fuel non-oxidative PPP for nucleotide synthesis [55]. Additionally, p53 inhibits the active dimerization of glucose 6-phosphate dehydrogenase, an essential PPP enzyme implicated in tumorigenesis through NADPH generation. Glucose 6-phosphate dehydrogenase is directly transactivated by HIF-1α and interacts with VEGF in cancer cells, linking PPP activation to angiogenesis [44]. Moreover, studies have suggested that enhanced PPP activity confers radioresistance to solid cancer cells [32].

### 3.3. HIF-1α-Induced Mitochondrial Dysfunction

The interaction between HIF-1α and mitochondrial dysfunction is a critical response to hypoxic conditions. Under hypoxic conditions, reduced mitochondrial function is observed, and dysfunctional mitochondria are enhanced. These dysfunctional mitochondria are cleared by targeted autophagy (mitophagy) [48]. This aligns with the Warburg effect, in which deregulated HIF-1α activity is observed along with mitochondrial impairment [31]. This leads to the impairment of essential proteins related to the TCA cycle, ETC, and broader mitochondrial respiration, collectively involved in a reduced metabolism [31]. This compromised mitochondrial function is not only implicated in cancer development but also triggers metabolic reprogramming within the cell [32]. However, the relationship between HIF-1α and mitochondrial dysfunction is a complex process. HIF-1α activation, often triggered by hypoxia, leads to a decrease in the mitochondrial oxidative metabolism, accompanied by an increase in glucose uptake and glycolysis [31]. This metabolic switch, known as the aerobic glycolysis phenotype, results in metabolic reprogramming, leading to mitochondrial dysfunction [55,56]. HIF-1α exerts a significant effect on mitochondrial function in solid tumors, contributing to metabolic reprogramming and potentially driving tumorigenesis. This influence is revealed through complex mechanisms that suppress mitochondrial activity and biogenesis, resulting in aerobic glycolysis [31,56]. HIF-1α directly affects mitochondrial protein levels. It upregulates miR-210, which specifically targets iron–sulfur cluster assembly protein 1/2, which is critical for the assembly of functional proteins in the ETC and TCA cycle, including aconitase [56]. The miR-210-mediated suppression of iron–sulfur cluster assembly protein 1/2 leads to the decreased activity of these key mitochondrial enzymes, thereby compromising OXPHOS and ATP production [57]. Furthermore, HIF-1α negatively regulates the oncogene c-Myc, thereby suppressing mitochondrial biogenesis [55]. The c-Myc promotes mitochondrial biogenesis by inducing the expression of peroxisome proliferator-activated receptor gamma coactivator-1α, an important regulator of mitochondrial function. Reduced Myc activity and HIF-1α effectively reduce the overall mitochondrial population within the cell [55]. These combined effects of HIF-1α on mitochondrial activity and biogenesis ultimately lead to a metabolic shift towards aerobic glycolysis, even under normoxic conditions (Warburg effect) [31]. This metabolic switch promotes tumor growth and progression by altering various cellular processes such as proliferation, invasion, and angiogenesis [32].

However, the precise mechanisms underlying this complex phenomenon remain unclear. By suppressing mitochondrial protein levels and biogenesis, it orchestrates a metabolic shift towards aerobic glycolysis, contributing to the Warburg effect and potentially fostering tumorigenesis. The specific molecular pathways and protein interactions that govern this HIF-1α–mitochondrial dysfunction relationship may be potential targets for new therapeutics. Thus, HIF-1α exerts a multifaceted influence on mitochondrial function in solid tumors. Understanding these intricate mechanisms holds immense potential for developing novel therapeutic strategies aimed at disrupting this pathological cascade and restoring mitochondrial function in patients with cancer.

### 3.4. HIF-1α’s Effect on Electron Transport Chain/ROS Production 

During OXPHOS, electrons derived from NADH and FADH2 are transported to the ETC and ultimately reduce oxygen in complex IV [31]. However, under hypoxic conditions due to limited oxygen availability, this homeostasis is disrupted. The lack of oxygen molecules inhibits complex IV formation, leading to electron accumulation within the ETC [48], which leads to electron leakage and the production of reactive oxygen species in the mitochondria [31]. The formation of reactive oxygen species (ROS) including superoxide, hydrogen peroxide, and hydroxyl radicals causes oxidative damage to proteins, lipids, and DNA, resulting in genomic instability and tumorigenesis [28]. In solid tumors, the ROS generation mediated by mitochondria is increased, which suppresses prolyl hydroxylase (PHD) and the factor-inhibiting hypoxia-inducible factor (FIH), leading to the accumulation and stabilization of HIF-1α [48]. Therefore, the elevated expression of HIF-1α promotes the expression of angiogenic genes, an altered glucose metabolism, and cell proliferation, thus promoting tumor growth and progression [54]. Therefore, hypoxia-induced mitochondrial dysfunction and ROS not only damage cellular components but also stabilize HIF-1α, which triggers downstream pro-tumorigenic-signaling pathways.

### 3.5. Activation of HIF-1α and Angiogenesis by Lactate Uptake 

Monocarboxylate Transporters (MCT) are responsible for the transportation of lactate. HIF-1α enhances the expression of MCT in the plasma membrane of tumor cells [49]. Lactate is transported via MCT-4 from tumor cells that lack fuel to feed other tumor cells [44]. Lactate is then taken up by cancer cells via MCT-1 and converted to pyruvate by the enzymatic activity of lactate dehydrogenase-B. The increase in intracellular pyruvate levels suppresses α-ketoglutarate (α-KG), which stabilizes and activates HIF-1α. This activation leads to the induction of VEGF-dependent tumor angiogenesis and the acceleration of tumor growth [32]. It has been suggested that a high amount of lactate (10mM) in cancer stimulates HIF-1α and angiogenic precursors such as VEGF [44].

Defects in carbohydrate metabolism pathways may also activate HIF-1α in cancer cells under normoxic conditions [32]. PHD and FIH-2 require substrate O_2_ as well as α kg as a co-factor for the hydroxylation of HIF-1α. Therefore, a decrease in intracellular α kg levels due to the overexpression of the α subunit of isocitrate dehydrogenase 3 (IDH3), IDH-3α, or mutations and resultant amino acid substitutions in succinate dehydrogenase or fumarate hydratase in the TCA cycle of cancer cells results in the unhydroxylation of P402, P564, and N803. The higher expression of IDH-3α mediates HIF-1α-induced metabolic reprogramming and angiogenesis [58,59].

## 4. Role of HIF-1α in Tissue and Matrix Remodeling 

The ECM serves as a vital scaffold and creator of cellular behavior within the TME. HIF-1α remodels ECM by depositing collagen and promoting fibrosis [26]. Under hypoxia, the elevated expression of HIF-1α in solid tumors causes ECM remodeling, which helps in tumor progression and metastasis [25]. HIF-1α upregulates collagen synthesis and promotes fibrotic ECM conditions, which allows a sequestering situation to occur in the immune system [25]. Simultaneously, it activates key MMPs such as MMP2, MMP9, and MMP15, which are responsible for ECM degradation and remodeling [25]. These MMPs, often overexpressed in tumors, act as molecular scissors, cleaving and altering the ECM and facilitating invasion and metastasis [27]. MMP9, in particular, plays a dual role. It not only modulates ECM remodeling but also fuels angiogenesis by influencing the “angiogenic switch” and releasing pro-angiogenic factors such as VEGF and FGF-2 [27]. This HIF-1α-mediated vascular remodeling results in a leaky vasculature within the TME, increasing plasma protein permeability, and promoting tumor growth and propagation [60]. For example, a hepatocellular carcinoma (HCC) requires epithelial mesenchymal transition (EMT) for tumor metastasis. EMT, a biological process that allows polarized epithelial cells, is promoted by hypoxia-induced HIF-1α activation [61]. This transition involves upregulating mesenchymal markers, such as fibronectin 1 and snail, while downregulating e-cadherin, leading to increased cell motility and invasive potential. The selective inhibition of HIF-1α by canagliflozin reverses EMT by altering protein expression and ROS levels [61]. Therefore, HIF-1α-mediated ECM remodeling is a critical feature that alters the overall TME, making it more prone to tumor formation, invasion, and metastasis [62]. Targeting HIF-1α to inhibit tumor progression could serve as a new therapeutic target.

### 4.1. HIF-1α-Induced Cell Proliferation and Differentiation

The relationship between HIF-1α and angiogenesis in solid tumors is a major player in cellular adaptation and pathological consequences. As discussed above, HIF-1α acts as a major player in orchestrating the expression of essential genes, which in turn promotes angiogenesis, a process that is essential for tumor cell growth and metastasis [9,10,11]. Angiogenesis is the de novo formation of blood vessels from the pre-existing vasculature [63]. This process is tightly regulated and maintained during cellular activities, with the balance of angiogenic and pro-angiogenic factors being known as the “angiogenic switch” [27]. However, under hypoxia, this equilibrium is disrupted in solid tumors. During hypoxia, HIF-1α is stabilized and expresses multiple genes that interrupt this angiogenic switch to perpetuate angiogenesis [11,24,63]. This HIF-1α-driven activation of pro-angiogenic factors such as VEGF disrupts the delicate balance of the angiogenic switch, causing new vessel formation [24]. The importance of angiogenesis in tumor growth and metastasis is well established. Muthukkaruppan et al.’s study in 1982 demonstrated the growth behavior of cells with and without access to blood circulation. This study highlights the dependence of tumor cells on newly formed blood vessels for their nutrient and oxygen supply, indicating that angiogenesis is critical in tumor progression [64,65].

Solid tumors face nutrient deficiency and an inadequate oxygen supply, and the activation of angiogenic stimulators is required for their survival. HIF-1α is the main activator of hypoxia-induced angiogenesis, as shown in Table 1. Therefore, understanding the mechanisms by which HIF-1α activates angiogenic stimulators is crucial for the development of effective therapeutic strategies. In this context, exploring the signaling cascades triggered by the HIF-1α-induced activation of key angiogenic stimulators, such as VEGF, holds immense potential. Therefore, the HIF-1α-induced activation of some important angiogenic stimulators was explored with the help of string analysis, which provides the signaling cascade [66]. The progression of the solid tumor is reliant upon angiogenesis and HIF-1α, both of which are critical factors. In light of this, we seek to offer a novel perspective on the inhibition of angiogenesis as a means to prevent the advancement of tumor progression. These insights may be utilized for the development of novel therapeutics that target the angiogenic switch.

#### 4.1.1. VEGF

VEGF, a key signaling protein within a family of ligands (A, B, C, and D), exerts a significant role in both vasculature development and disease processes [67,68,69,99]. Its specific interaction with endothelial cell surface receptors (VEGFR-1, -2, and -3) orchestrates diverse cellular responses [Table 2]. Specifically, VEGF is the most potent, known endothelial cell mitogen and plays an important role in vasculogenesis and pathological angiogenesis [65,68]. Hypoxia triggers a cascade of gene expression involving HIF-1α and VEGF via the mitogen-activated protein kinase (MAPK) pathway. HIF-1α binds to the hypoxia-response element (HRE) within the VEGF promoter, significantly enhancing its transcriptional activity [70,100,101,102]. The HIF-1α/HRE complex directly upregulates VEGFR-1 expression in tumor cells, further amplifying VEGF signaling [102]. Clinical studies have revealed a strong correlation between elevated HIF-1α and VEGF levels and decreased treatment efficacy and patient survival [71].

**Figure 3:**
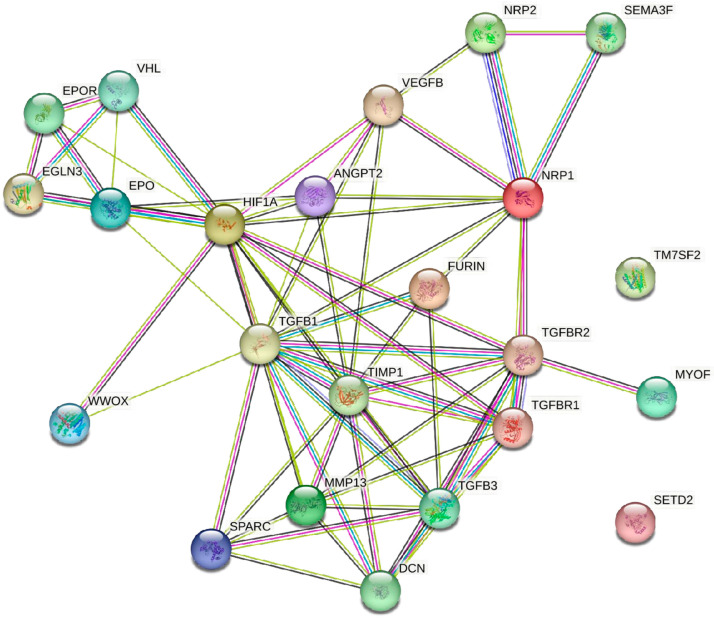
String analysis data to represent the signaling cascade of HIF-1 with angiogenic stimulators. Abbreviations: ANGPT 2: angiopoietin 2; DCN: decorin; EPOR: erythropoietin receptor; EPO: erythropoietin; EGLN3: elegans gene egl-9 homolog 3; HIF1A: hypoxia-inducible factor 1 alpha; MMP13: matrix metalloproteinase 13; MYOF: myoferlin; NRP: neuropilin; SEMA3F: semaphorin-3F; SEDT2: SET Domain Containing 2; SPARC: secreted protein acidic and rich in cysteine; TGFB: transforming growth factor beta; TGFBR: transforming growth factor beta receptor; TIMP1: tissue inhibitor matrix metalloproteinase 1; TM7SF2: transmembrane 7 Superfamily Member 2; VEGFB: vascular endothelial growth factor B; VHL: Von Hippel–Lindau tumor suppressor protein; WWOX: WW domain containing oxidoreductase.

Findings from string analysis (Figure 3) also support this relationship, where HIF-1α is found to have a direct connection with VEGF. Similarly, a report on the synergistic relationship between these two factors showed that HIF-1α stabilization under hypoxia potently activates VEGF expression [102]. This synergistic effect of HIF-1α on VEGF results in increased mRNA levels and overexpression compared to other proteins in tumor cells [100]. Interestingly, therapeutic efforts targeting HIF-1α have demonstrated promising results in reducing VEGF-mediated tumor progression. In liver cancer models, the antidiabetic drug canagliflozin effectively reduced VEGF-A expression by inhibiting HIF-1α under hypoxic conditions [61]. Similarly, everolimus, an mammalian target of the rapamycin (mTOR) inhibitor and a positive regulator of HIF-1α, downregulated both VEGF-A and VEGF-C levels in head and neck squamous cell carcinoma studies, both in vitro and in vivo [72]. These findings suggest that targeting HIF-1α indirectly suppresses VEGF and its downstream effects, potentially hindering tumor progression and lymph angiogenesis.

The proangiogenic effects of HIF-1α-induced VEGF extend beyond direct endothelial cell activation. VEGF facilitates vascular permeability, stimulates endothelial cell growth and proliferation, and forms complex networks with other cytokines and growth factors, collectively contributing to robust tumor progression [48]. For instance, its interactions with FGF and PDGF further support angiogenesis and cell development [24]. Additionally, VEGF interacts with the epidermal growth factor receptor pathway to promote tumor cell proliferation and survival, highlighting its multifaceted influence on tumor biology. Therefore, the HIF-1α-driven VEGF-signaling cascade is a critical driver of tumor angiogenesis.

VEGF activation by HIF-1α suppresses dendritic cell expression while enhancing the recruitment of myeloid-derived suppressor cells and T-regulatory cells. Thus, creating an immunosuppressive TME that prevents tumor cells from undergoing cytotoxicity allows the tumor cells to survive and expand [103]. Under hypoxic conditions, tumor-associated macrophages further produce VEGF and stimulate MMP-9 secretion, collectively promoting tumor angiogenesis and invasive potential [104]. Furthermore, hypoxic conditions elevate intracellular pyruvate levels, which inhibits the 2-oxoglutarate formation to stabilize HIF-1α. This stabilization promotes an increased level of VEGF expression and the subsequent activation of angiogenesis [105]. As discussed in Section 3.2, p53 is downregulated by the overexpression of HIF-1α due to the hypoxic TME. Recent studies suggest that p53 mutations cause the sustained activation of the mTOR pathway, which is a positive regulator of HIF-1α [62,72].

#### 4.1.2. Relation of HIF-1α, p53, VEGF, and MMPs in Tumor Angiogenesis

String analyses revealed that HIF-1α, p53, VHL, and PHD2/3 share common pathways (Figure 3). The roles of these proteins in tumorigenesis and angiogenesis have been reported in several studies [62,103]. Under hypoxic conditions, the inactivation of these dioxygenases leads to p53 inactivation or mutation and finally stabilizes HIF-1α [106]. HIF-1α induces VEGF expression, which plays a central role in angiogenesis by activating other pro-angiogenic factors and regulating endothelial proliferation [106]. It activates other pro-angiogenic factors and regulates endothelial proliferation, serving as a central modulator of the entire process [100,106,107]. This crucial role is further supported by clinical findings in colorectal cancer, where HIF-1α positively correlates with VEGF and is associated with an advanced tumor stage and metastasis [108]. HIF-1α, VEGF, and MMPs form a synergistic trio that culminates in capillary development and angiogenesis under hypoxic conditions [26]. The selective inhibition of HIF-1α by indolephenoxyacetamide promotes the inhibition of MMP-2 and MMP-9, and consecutively reduces the angiogenesis precursor VEGF-A [106]. This dual action not only suppresses angiogenesis but also upregulates p53, highlighting the potential therapeutic synergy. However, the inhibition of the VEGF pathway has been considered as a first-line treatment to prevent angiogenesis in solid tumors for decades and has shown limitations. Several studies have reported treatment failure or even disease progression attributed to the emergence of compensatory pathways upon VEGF inhibition [73]. Probably, the reason behind this is the involvement of alternative pathways in angiogenesis after the inhibition of VEGF, which compensates for its effect on VEGF for angiogenesis. This underscores the need for a more nuanced approach that considers the complex network of angiogenic factors beyond VEGF alone. These complex mechanisms underlying the interactions between HIF-1α, p53, VEGF, and MMPs are crucial for developing effective therapeutic strategies. Targeting multiple variables within this network can potentially disrupt the angiogenic process and impede tumor progression, offering a more promising approach for treating solid tumors.

#### 4.1.3. PDGF

PDGF and its receptors (PDGFRs) have been shown to promote tumor growth and invasion by exerting effects on tumor cells and their surrounding microenvironment [74]. PDGF is composed of four isoforms (PDGF-A, PDGF-B, PDGF-C, and PDGF-D) and forms a single polypeptide unit that can generate five functional homo- or heterodimers, PDGF-AA, PDGF-BB, PDGF-AB, PDGF-CC, and PDGF-DD, upon binding to PDGFR-α and PDGFR-β [73]. Through dimerization and phosphorylation, PDGF ligands increase the number of binding sites for subsequent signaling [75]. PDGF is involved in numerous physiological processes, including embryonic development, wound healing, and the maintenance of interstitial fluid pressure in tissues (Table 1) [76].

HIF-1α also induces PDGFR expression, which facilitates angiogenesis [77]. However, hypoxia can upregulate the expression of PDGF-BB and PDGFR-β, which are tightly associated with the hypoxia marker HIF-1α [109]. The activation of the PI3K/AKT-signaling cascade by the extracellular binding of PDGFR to its ligands, PDGF-A and PDGF-B, leads to an increase in HIF-1α transcriptional activity [77]. The activation of these receptor tyrosine kinases has been associated with HIF-1α-mediated angiogenesis, which also activates other important pathways, including the janus kinase/signal transducers and activators of transcription (JAK/STAT), extracellular signal-regulated kinase (ERK), MAPK, AKT, and Notch pathways [75,95]. It was found in osteosarcoma that the hypoxia-upregulated PDGF-BB/PFGFR axis promotes cell proliferation and migration. Furthermore, it markedly enhances the phosphorylation of AKT, ERK1/2, and STAT3 [109]. Therefore, the PDGF/PDGFR signaling pathway is recognized as one of the most important receptor tyrosine kinase pathways in cancer, as it is involved in proliferation, migration, invasion, metastasis, and angiogenesis, all of which contribute to the growth of solid tumors [75].

PDGFRs’ expression is known to vary under physiological and pathological conditions. For instance, PDGF-α tends to be found in tumor cells, whereas PDGF-β is commonly observed in stromal and perivascular cells [74]. These growth factors are often co-expressed in a paracrine manner, but they also have an autocrine effect on cell motility and the proliferation of tumor cells, which aids tumor growth [74,76]. In addition, tumor cells secrete PDGF through a paracrine pathway to influence non-tumor cells such as immune cells, stromal fibroblasts, and tumor blood vessels, thereby promoting tumor development, angiogenesis, and the formation of a favorable TME [74,76]. In addition to its proliferative and chemotactic actions on lymphatic endothelial cells, HIF-1α also facilitates the lymphatic metastasis of breast cancer. It has been proposed that the PDGF-B signaling in lymph angiogenesis is induced by the paracrine impact of HIF-1α. However, a decreased lung metastasis and lower peritumoral lymphatic vessel density were caused by the genetic deletion of HIF-1α and PDGF-B. [110]. Therefore, a strong linear association was observed between HIF-1α and PDGF-B/PDGFR-β. This suggests that reducing initial tumor development and lymphatic dissemination could be a potential strategy to lower tumor metastasis and mortality.

HIF-1α directly upregulates VEGF and its influence cascades through the PDGF-BB activation of stromal fibroblasts [111]. These activated fibroblasts become hubs for generating PDGF-CC, EPO, and FGF, collectively promoting VEGF-independent tumor angiogenesis [74]. This coordinated network expands beyond PDGF-BB, encompassing various pro-angiogenic molecules, such as ANG 1, ANG 2, ANGPTL1, and EPO (Figure 3). Consequently, the overstimulation of PDGF, in isolation or in synergy with VEGF and FGF, promotes malignant vasculature [73]. Moreover, PDGF stimulates the secretion of FGF-2 to promote angiogenesis [112]. HIF-1α-positive glioblastoma tumors exhibit a significant co-localization of VEGF and PDGF-C within their cells [113], highlighting a strong correlation between these factors and HIF-1α activity. This colocalization extends beyond glioblastoma, as Clara et al. (2014) observed, similar to the cytoplasmic co-expression of VEGF and PDGF-C in other tumor cell populations. In a preclinical study, rat xenograft models demonstrated that tyrosine kinase inhibitors effectively curtail ligand-induced PDGFR activation, leading to reduced tumor growth and micro vessel density [76]. PDGF inhibition synergizes with VEGFR-targeting drugs, yielding more pronounced antitumor effects. Importantly, the resistance to anti-VEGF therapy in recurrent glioblastoma has been linked to elevated PDGF-C and c-MET expression, highlighting the potential of PDGF-targeting as a complementary strategy [113]. HIF-1α-PDGF-VEGF represents a multifaceted network that drives tumor angiogenesis; therefore, the simultaneous targeting of VEGF and PDGF could offer more effective and comprehensive approaches to combat tumor progression.

#### 4.1.4. FGF

The fibroblast growth factors are a family of proteins involved in a variety of cell signaling pathways involved in proliferation and growth. The FGF family is comprised of 23 members, although there are only 18 FGFR ligands [114]. When a FGF binds to a specific FGFR, it causes the receptor to dimerize and activate its intracellular domain. This leads to the phosphorylation of downstream signaling molecules, which in turn activates the ERK/MAPK pathway by increasing kinase activity [115]. Therefore, the FGF pathway plays a crucial role in various biological processes such as tissue repair, embryonic development, and other cellular functions (Table 1) [116]. Stimulation of HIF-1α increases FGF expression [78]. Stabilization of HIF-1α increases FGFR3 mRNA and protein levels in bladder cancer. However, the mRNA level of FGFR3 was considerably reduced by downregulating HIF-1α, but not HIF-2α. This indicates that the regulation of the FGF and FGFR is dependent on HIF-1α [117]. The disruption of FGF signaling promotes tumor growth by increasing angiogenesis and metastasis [79]. Additionally, it stimulates the secretion of VEGF and promotes the sprouting and branching of endothelial cells [78]. Elevated FGF levels have been observed in various solid tumors, including breast, lung, bladder, and prostate cancers [79]. For example, FGFR3 expression was elevated in hypoxia and HIF-1α-dependent manners in bladder cancer [117]. Another member of the FGF family, FGF11, has been implicated in carcinogenesis, specifically in the invasion and growth of tumors. HIF-1α was discovered to regulate FGF11 by binding to the FGF11 promoter region, which in turn promotes FGF11 in hypoxic conditions. According to Yang et al. (2015), the overexpression of FGF11 may help stabilize capillary-like tube formation, but it is not involved in cell migration [118]. A recent study on thyroid cancer found that FGF11 knockdown prevents hypoxia-induced tumor cell invasion, migration, and proliferation. Consequently, FGF11 and HIF-1α establish a positive feedback loop that aids in the development and spread of thyroid cancer [119].

FGF signaling has been shown to impact fibroblast activity and induce phenotypic changes in fibroblasts, ultimately resulting in the formation of cancer-associated fibroblast (CAF). CAFs modulate the activity of growth factors and chemokines, attract tumor-associated macrophages, and play a role in the EMT pathway. The activation and stimulation of FGF and CAF promotes tumor formation, growth, and invasion [25,80]. FGF releases growth factors, MMPs, and other cytokines that alter the levels of collagen and fibronectin, resulting in a dense and fibrotic ECM that promotes cell survival, invasion, angiogenesis, and protects cancer cells from the immune system by creating an immune-suppressive TME [81]. In earlier research on prostate cancer, it was noted that CAF upregulates HIF-1α to increase malignancy and enhance invasion, metastasis, and antitumor efficacy [82]. Similar results were found in an HCC study, where CAF-derived CCL5 inhibited ubiquitination degradation, and HIF-1α expression under hypoxia promoted EMT and lung metastasis through the activation of zinc finger E-Box-binding homeobox 1 (ZEB1). The poor prognosis and metastasis of HCC are brought on through this CAF-derived CCL5 mediated HIF1α/ZEB1. Consequently, it is possible that CAFs influence angiogenesis by promoting HCC cell invasion and metastasis in the TME [120]. However, FGF controls the role of transcriptional factors and downstream signaling molecules in the EMT; this transition leads to therapeutic resistance and promotes invasion and metastasis. This signaling pathway also modulates the metastatic environment and promotes tumor cell colonization in various organs [81]. The activation of FGF signaling inhibits the recruitment of immune cells, such as macrophages and T cells, and enhances the recruitment of immunosuppressive and tumor-promoting cytokines and chemokines, as discussed above. The FGF also promotes cell cycle progression and proliferation by increasing the production of cell cycle regulatory proteins, including cyclin D1, and improving their interaction with cyclin-dependent kinases [121]. This suppresses the production of cyclin-dependent kinase inhibitors, such as p21 and p27, to promote cell cycle progression. However, the FGF phosphorylates the retinoblastoma protein, another critical regulator of phase transition (G1/S), to regulate the cell cycle [81,122].

The complex relationship between FGF signaling and tumorigenesis has garnered significant attention in cancer therapy. Emerging evidence suggests that the dysregulation of FGF signaling not only promotes tumor growth and progression but also contributes to the development of resistance to established treatment modalities. Studies have demonstrated that activated FGF signaling in tumor cells can confer resistance to therapies targeting VEGF, a key mediator of tumor angiogenesis [78,123]. However, a paradigm shift has emerged in the exploration of combined therapeutic strategies. The synergistic application of FGF inhibitors along with anti-VEGF therapy has shown promising results in preclinical models, leading to reduced vascular density and a restored tumor sensitivity to treatment [78,123]. This synergistic effect highlights the potential of disrupting multiple pro-tumorigenic pathways to enhance therapeutic efficacy.

In addition to VEGF-targeted therapies, the FGF signaling blockade has demonstrated efficacy in other treatment contexts. Studies focusing on mammary carcinoma models have reported that inhibiting FGF signaling not only hinders tumor growth but also downregulates VEGF-C expression [124]. This downregulation translates to improved overall survival, further solidifying the potential of FGF-targeted therapies in various cancer types. These findings collectively underscore the multifaceted role of FGF signaling in a cancer’s progression and resistance to therapy. Dysregulated FGF signaling poses a challenge for chemotherapy and targeted therapy and can act as a “bypass mechanism” allowing tumor cells to evade treatment effectiveness [125]. Therefore, targeting FGF signaling has emerged as a promising approach to overcome resistance mechanisms and optimize cancer therapeutic strategies. Further research exploring the complex interactions between FGF signaling and other therapeutic modalities is crucial to fully unlock its potential in clinical practice.

#### 4.1.5. TGF-β

TGF-β is an epidermal growth factor that exists in three isoforms: TGF-β1, TGF-β2, and TGF-β3 [83]. It is the most extensively studied pathway for the EMT signaling pathway, which includes the HIF-1α-related suppressor of mothers against decapentaplegic homologs’ (SMADs’) signaling and non-SMAD signaling [84]. In the case of SMAD signaling, TGF-β interacts with its receptors, TGFR I and II, and activates the downstream mediators SMAD2 and SMAD3. The oligomerization of SMAD2/3 with SMAD4 leads to their migration towards the nucleus, where they regulate gene expression [83]. Non-SMAD signaling, on the other hand, involves various MAPKs, PI3K/AKT, and mTOR [83,84]. The TGF-β-signaling framework is crucial for governing cell proliferation, differentiation, tissue repair, and carcinogenesis (Table 1) [85,86]. However, TGF-β promotes the stability of HIF-1α by suppressing the mRNA and protein expression of PHD2 [84].

TGF-β1 is the primary isoform expressed in the immune system and lymphoid organs, where it is essential for T-cell homeostasis, T-regulatory cells, and effector cell functions [83]. In healthy tissues, TGF-β functions as a tumor suppressor and growth inhibitor [86]. Nevertheless, during tumor progression, cancer cells develop a resistance to TGF-β to avoid growth inhibition signals. Consequently, during tumor growth, TGF-β functions as a tumor promoter instead of tumor suppressor [87]. Hypoxia and TGF-β together stimulate VEGF mRNA levels in fibrosarcoma through an activator protein-1/HIF-1α-dependent pathway, potentially amplifying the hypoxic response [126]. This alteration may be due to the activation of HIF-1α, which regulates the TGF-β-SMAD3 pathway [84].

The interaction between HIF-1α and TGF-β signaling transcends a simple linear interaction, instead resembling a complex and synergistic process within the TME. This interaction plays the most significant role in tumor progression and immunosuppression. HIF-1α upregulates the expression of various immunosuppressive molecules in tumor cells, including galectin-9 (Gal-9) [84]. Gal-9 dampens antitumor immune responses, allowing tumor cells to evade immune surveillance [85]. In the early stages of tumor growth, HIF-1α induces TGF-β via an autocrine loop, activating Gal-9 [127]. While TGF-β initially exhibits tumor-suppressive potential by inhibiting T-cell activation and antigen presentation [86], its excessive release by tumor cells creates an immunosuppressive TME [87]. Later in tumorigenesis, TGF-β activates its own expression through SMAD3, perpetuating an immunosuppressive environment [127]. This TGF-rich TME attracts and activates fibroblasts, further promoting tumor invasion [128]. The SNAI1-SMAD3/4 complex, activated by TGF-β, drives EMT, a key step in metastasis [129]. Additionally, TGF-β suppresses E-cadherin expression and promotes EMT via TWIST activation [130]. Both HIF-1α and TGF-β actively regulate angiogenesis, which is crucial for tumor growth and metastasis [102]. Their cooperation amplifies their individual effects, fostering a pro-tumorigenic vasculature. This intertwined signaling remodels the TME to favor tumor cell survival and growth. Hypoxia itself influences TGF-β expression, further promoting HIF-α and TGF-β interaction [85,131]. This interaction between HIF-1α and TGF-β creates a favorable TME condition for tumor progression. Therefore, this interaction regulates immunosuppression, EMT induction, and angiogenesis, resulting in metastasis and a poor clinical outcome. Targeting this interaction may provide an alternative therapeutic potential for disrupting tumor progression.

#### 4.1.6. ANG 1 and ANG 2

ANGs, including ANG 1, ANG 2, ANG 3, and ANG 4, are crucial facilitators of angiogenesis. Their interaction with the tyrosine kinase receptors Tie1 and Tie2 orchestrates vessel growth, stability, and maturation. ANG 1, a potent angiogenic growth factor, binds to Tie2 and promotes endothelial cell survival, vascular stability, and barrier function [88,89]. It acts as a paracrine hormone secreted by mesenchymal cells, activating downstream signaling pathways, such as Akt, to inhibit the transcription factor forkhead box protein O1 and downregulate ANG 2, its antagonist [90]. ANG 1 itself is not a mitogen, but it synergizes with VEGF and amplifies its pro-angiogenic effects and promotes increased vascular branching [89,132]. In contrast, ANG 2, which is expressed by endothelial cells, exhibits complex duality. Although it can weakly activate Tie2, its primary function lies in antagonizing ANG 1–Tie2 signaling, acting as a “anti-vascularizing agent” [68]. Hypoxia and VEGF are known to upregulate ANG 2, suggesting their potential role in remodeling the tumor vasculature [91,92]. Previously, it was believed that HIF-1α directly activates angiopoietins. Instead, HIF-1α does not directly induce ANG 1 or ANG 2, whereas under hypoxic conditions, HIF-1α upregulates VEGF, which subsequently stimulates the production of both ANG 1 and ANG 2 (Figure 3) [89]. This coordinated action promotes angiogenesis, particularly in solid tumors, where elevated levels of HIF-1α, VEGF, and ANG 2 are observed [89,132]. Arsenic trioxide, an anticancer agent, disrupts the HIF-1α–VEGF–ANG axis by inhibiting PI3K-Akt/HIF-1α signaling, thereby limiting the paracrine activity of ANG 1 and ANG 2 [133], resulting in the inhibition of angiogenesis and tumor growth. However, this effect can be partially reversed by overexpressing HIF-1α; HIF-1α plays an important role in ANG 1 and ANG 2 expression in solid tumors.

In HCC, ANG 1 and ANG 2 play distinct, yet intertwined roles in regulating vascularity. ANG 1, a potent Tie2 receptor agonist, promotes vessel maturation and stabilization by recruiting pericytes and smooth muscle cells (Table 1) [89]. In contrast, ANG 2, known for its vascular remodeling properties, is expressed at sites of dynamic vessel changes, the loosening of the endothelium, and the facilitation of pro-angiogenic VEGF access [93]. The interaction between these angiopoietins is crucial. While ANG 2 overexpression can competitively block ANG 1–Tie2 binding, destabilizing the vasculature, and exposing endothelial cells to VEGF’s pro-angiogenic signals [93], it can also synergize with VEGF under specific conditions [94]. This complex process dictates the fate of the nascent vessels, potentially leading to their maturation or regression. Furthermore, HIF-1α, a key player in HCC progression, induces VEGF expression, triggering endothelial cell migration, proliferation, and sprouting, ultimately promoting angiogenesis [67,101]. Elevated VEGF and ANG 2 levels can facilitate excessive and potentially disorganized angiogenesis, whereas reduced ANG 2 and increased ANG 1 levels can lead to the stabilization and maturation of new vessels, potentially impacting tumor progression and therapeutic efficacy [134]. Supporting this notion, studies have demonstrated that suppressing HIF-1α in HCC significantly reduces cell proliferation and invasiveness, highlighting the potential therapeutic benefits of targeting this pathway for therapeutic benefits [132].

Increasing evidence paints a fascinating picture of the dual nature of the ANG family in cancer, highlighting their potential as both therapeutic targets and prognostic biomarkers. Studies have revealed significantly elevated plasma levels of ANG 2 compared to ANG 1 in patients with cancer [94]. This altered ANG 1/ANG 2 expression disrupts their delicate balance, tipping the scales in favor of ANG 2. The consequent increased ANG 2–Tie2 binding promotes vascular instability, culminating in tissue hypoxia and triggering the “angiogenic switch” [94]. This actively encourages abnormal angiogenesis, often gravitating towards the tumor margin, regulated by the interaction of VEGF and ANG ½–Tie2 signaling cascades [94]. In another study, patients with higher VEGF and ANG 2 levels in multiple myeloma exhibited poorer prognoses, suggesting a synergistic pro-tumorigenic effect [134]. HIF-1α can induce angiopoietin-like proteins (ANGPTL) such as ANGPTL 2 and ANGPTL 4, further adding to the pro-tumorigenic effect. These HIF-1α-induced factors have been implicated in facilitating proliferation, metastasis, angiogenesis, and glycolysis in osteosarcoma [9,24]. Collectively, these findings reveal the critical role of ANGs in shaping the TME.

#### 4.1.7. EPO

EPO is a regulatory hormone that facilitates the formation of red blood cells, plays an essential role in oxygen transport, is enhanced under hypoxia, and promotes erythropoiesis (Table 1) [10,96]. EPO and its receptor, EPOR, activate signaling cascades such as JAK2/STAT5, MAPK, and PI3K/AKT, which are interconnected and crucial for cell survival, and exhibit pleiotropic effects on various cell types and tissues [97]. It is widely accepted that HIF-1α stimulates EPO production under hypoxic conditions. This is due to the fact that HIF-1α was initially discovered for its transcriptional activity after being implicated in the regulation of EPO and the induction of hematopoiesis [32].

The interaction between EPO and HIF-1α is a well-established pathway that promotes angiogenesis in response to low oxygen availability. Under hypoxic conditions, HIF-1α activates EPO transcription, leading to increased EPO expression. This, in turn, synergizes with VEGF to stimulate the growth of new blood vessels, aiming to restore oxygen delivery to deprived tissues [135]. Studies have demonstrated that both EPO and VEGF exhibit potent proangiogenic activity, promoting endothelial cell proliferation, migration, and tube formation [97]. The inhibition of HIF-1α leads to the downregulation of EPO mRNA levels, further supporting this notion, suggesting that HIF-1α acts as a crucial upstream regulator of EPO expression under hypoxia [135]. However, the precise mechanisms governing this pre-transcriptional regulation remain unclear.

The HIF–PHD–EPO signaling pathway plays a complex and context-dependent role in tumorigenesis (Figure 3). Under Hypoxia, PHD enzymes, regulated by EGLN3 activity, are inhibited, leading to the accumulation and activation of HIF-1α [95]. This activated HIF-1α, particularly in renal fibroblasts, upregulates EPO production, stimulating red blood cell production, and potentially improving oxygen transport [10]. While increased EPO levels may initially enhance oxygen delivery via increased red blood cells, tumor cells can develop functional EPOR [136]. EPO stimulation through these receptors leads to increased VEGF expression, promoting angiogenesis and tumor growth [136]. Under hypoxic conditions, tumor cells can shift their metabolism towards glycolysis, producing lactate instead of ATP through oxidative phosphorylation, suggesting that the HIF–PHD–EPO interaction might influence tumorigenesis [98]. While some studies have suggested that EPO and EPOR promote tumorigenesis in adult and pediatric cancers, others have suggested their tumor-suppressive roles [136]. This highlights the need for further research to understand the nuanced and context-dependent effects of this pathway on different cancer types and stages. The involvement of EPO in cancer development is more complex, involving potential pro- and anti-tumorigenic effects depending on the specific tumor types [136]. Further research is crucial to unraveling these complexities and identifying potential therapeutic targets within this complex pathway.

##### EPO/EPOR in Adult Cancers: A Double-Edged Sword

In adult malignancies, such as breast and uterine cancers, EPOR expression is frequently observed. The uterine endometrium exhibits estrogen-mediated responsiveness to EPO, suggesting its potential role in hormone-sensitive tumors [137]. Moreover, increased EPOR expression in malignant breast tumors compared to their benign counterparts underscores its potential relevance in disease progression. Interestingly, suppressing EPO signaling in the tumor xenografts of both uterine and breast cancers leads to tumor and endothelial cell death, resulting in tumor size reduction and apoptosis [137].

##### EPO/EPOR in Pediatric Cancers: A Delicate Balancing Act

Pediatric cancers often have developmental origins, making the role of EPO/EPOR even more intriguing. As EPO and EPOR are crucial for embryonic development, their potential involvement in pediatric tumors warrants further investigation. Studies have shown that hypoxia-induced EPO can increase the expression of anti-apoptotic proteins such as mcl-1, bcl-xL, and bcl-1 in pediatric cancer cell lines through enhanced NF-κB activity [136]. This suggests that besides its role in erythropoiesis, hypoxia-induced EPO acts as a survival factor for pediatric cancer cells by promoting angiogenesis and inhibiting apoptosis.

The convergence of HIF-1α and EPO/EPOR signaling further underscores their complex influence on cancer progression [96,136]. During hypoxia, HIF-1α upregulates EPO expression, creating an autocrine loop that promotes angiogenesis and cell survival in tumors [96]. This complex interaction exemplifies the multifaceted roles of these factors, potentially acting as both tumor enablers in adult cancers and survival mechanisms in pediatric malignancies. Researchers have found that the increased expression of EPOR may promote cancer cell survival through bcl-2 and reduce the effectiveness of chemotherapy with cisplatin. These findings suggest that targeting the EPOR-signaling pathway might be a valuable strategy to improve treatment outcomes and combat therapy resistance in cancer [96,135,136].

## 5. Understanding the Coordinated Network among the HIF-1α and Angiogenic Stimulators 

Oxygen is a vital component of both normal and malignant cells as it enables the production of a significant amount of energy in the presence of glucose [49]. In non-tumor cells, under hypoxic conditions, the expression of hypoxia-responsive genes increases to alleviate chronic conditions such as ischemia, osteoarthritis, tendinopathy, and fibrosis [138,139]. Hypoxia-responsive genes, including those encoding EPO, VEGF-A, and glycolytic enzymes, are regulated by HIF-1α [139]. However, under normoxic conditions, HIF isoforms are inhibited by PHD and FIH and degraded via the proteasome degradation pathway [140]. Normal cells cease to divide in the absence of oxygen, whereas tumor cells continue to divide [49]. However, tumor cells continue to grow in hypoxic environments due to HIF-1α-regulated metabolic reprograming [140]. HIF-1α is known to regulate the expression of genes essential for cell survival under hypoxic conditions. HIF-1α is indispensable for the expression of glycolytic genes, including GLUT-1, VEGF-A, and LDH-A, whereas HIF-2α shows a limited impact and is often inhibited by HIF-3α [141]. HIF-1α stands out as the predominant regulator in most tumors, demonstrating a broader impact on hypoxic gene induction than HIF-2α [140,142,143,144,145]. Further studies highlight that HIF-1α, rather than HIF-2α, is essential for hypoxia-inducible gene responses in various cell lines, emphasizing its significance in solid tumors. The VEGF regulation by HIF-2α is complex, with conflicting results based on experimental approaches. RNAi techniques affirm HIF-1α’s role as the primary regulator of VEGF induction in different cell lines [146].

The interaction between HIF-1α and mitochondrial failure highlights the intricate nature of cellular responses to oxygen deprivation. The activation of HIF-1α triggers a range of downstream effects, including angiogenesis through lactate uptake, the modulation of ETC and ROS production, and the activation of PPP [48,49]. These complex pathways illustrate the diverse and interconnected influence of HIF-1α on the cellular metabolism, proliferation, and differentiation. HIF-1α plays a central role in the induction of VEGF expression under hypoxic conditions. The binding of HIF-1α to VEGF-HRE enhances VEGF transcription, leading to downstream signaling pathways that promote angiogenesis. The synergistic effects of HIF-1α on VEGF, as evidenced by string analysis data, underscore its crucial role in increasing mRNA levels and expression in tumor cells. VEGF’s interactions with other growth factors, such as FGF, PDGF, and EGFR, highlight its position as a central modulator in the complex network of angiogenesis (Figure 3) [24,101,103]. Although HIF-1α does not directly activate or induce ANG levels, it is a crucial regulator of VEGF. The interaction between ANG 1 and ANG 2 is finely tuned by HIF-1α-induced VEGF, with ANG 1 promoting vascular stability and maturation, while ANG 2 is involved in vascular remodeling. The altered expression of ANG 1/ANG 2, influenced by HIF-1α-induced VEGF, marks the initiation of the “angiogenic switch,” contributing to aberrant angiogenesis in cancer patients [89,93,94]. HIF-1α plays a vital role in EPO regulation under hypoxic conditions. The signaling cascades activated by EPO, facilitated by HIF-1α, contribute to cell survival and exert pleiotropic effects. EGLN3, identified through string analysis, has emerged as a potential therapeutic target for modulating HIF-1α function and impeding cancer progression. Additionally, HIF-1α induces PDGFR, which links PDGF to angiogenesis. The PDGF/PDGFR-signaling pathway intertwines with other angiogenesis-promoting molecules, emphasizing its significance in cancer development [73,77]. The HIF-1α stimulation of FGF signaling plays a critical role in embryonic development, tissue repair, and cancer progression. The activation of FGF signaling in tumor cells not only enhances angiogenesis, but also induces the formation of CAF. This dynamic interaction shapes the TME by altering the ECM composition and creating an immunosuppressive milieu. Targeting FGF signaling is crucial for cancer therapy, given its role in therapeutic resistance and evasion mechanisms [78,81,116,125]. TGF-β plays dual roles in cancer progression, acting as a tumor suppressor in healthy tissues and a tumor promoter in advanced malignancies. HIF-1α’s influence on TGF-β signaling highlights its contribution to EMT and immunosuppressive TME. The intricate cross-talk between HIF-1α, TGF-β, and other signaling pathways underscores the complexity of cancer progression and challenges associated with targeted interventions [83,87]. HIF-1α is a key player in the development of solid tumors [35], and its involvement in angiogenesis, glycolysis, and matrix remodeling has been well identified as a transcriptional master regulator [9,10,11]. The complex mechanisms involved in the development of solid tumors in response to hypoxia and HIF-1α play a significant role in the alteration of cellular processes. The role of HIF-1α in the remodeling of ECM, through the regulation of MMPs and collagen biosynthesis, plays a critical role in the alteration of TME homeostasis [25,26,27,49,62]. Moreover, the angiogenic switch regulated by HIF-1α is crucial for tumor growth and TME integrity. Targeting HIF-1α and angiogenic signaling networks may offer an effective therapeutic strategy [73,83,87].

## 6. Future Perspective

HIF-1α is widely recognized as a key target for the diagnosis and treatment of solid tumors because of its role in the progression of solid tumors [147]. Malignant solid tumors are typically treated with a combination of radiotherapy, chemotherapy, surgery, or targeted therapies. However, the effectiveness of these treatments is significantly reduced in a hypoxic TME [55]. Under hypoxic conditions, solid tumors rely on glycolysis for energy production, resulting in an acidic microenvironment that makes them more resistant to chemotherapy and radiotherapy. Despite this, it promotes the development of an aggressive TME [55]; therefore, it is necessary to develop novel HIF-1α inhibitors to improve antitumor therapy.

## 7. Conclusions

This review is an attempt to explore the role of HIF-1α and angiogenic proteins and their coordinated network in solid tumors. Hypoxia promotes malignant progression through various mechanisms, including the increased expression of transcription factors and gene products involved in tumor progression and the induction of genomic instability. HIF-1α is a key regulator of angiogenesis and glycolysis that supports tumor cell survival, proliferation, invasion, and metastasis. Hypoxia also fosters an immune-suppressive milieu that influences anti-tumor effects by secreting and activating immunosuppressive cells, such as T-regulatory cells, which leads to the inhibition of immune cells such as CD8 T-cells or cytotoxic T cells. HIF-1α-mediated programmed death cell ligand 1 expression has been observed in tumor cells, dendritic cells, and macrophages. Programmed death cell ligand 1 in cancer binds with programmed cell death protein 1 on CD8+T cells, inactivating CD8-T cells and preventing them from exerting cytotoxic effects on tumor cells. Hypoxia in tumor cells suppresses apoptosis, which is a natural process in normal cells. The inhibition of HIF-1α stabilization is the primary target for preventing modifications in cellular physiology. The activation of PHD2 can regulate the interaction between HIF-1α and solid tumors. FIH can also be considered as the target since it is more stable than PHD2 and can regulate HIF-1α even amidst low-oxygen availability. The Von Hippel–Lindau tumor suppressor regulates HIF-1α, but our focus is on the PHD family, as they are intermediaries between HIF-1α and the Von Hippel–Lindau tumor suppressor. Therefore, targeting HIF-1α and its related proteins is an effective approach for developing new anticancer therapeutics.

## Figures and Tables

**Table 1:** List of important angiogenesis stimulators and their specified roles.

Angiogenesis Stimulator	Role in the Angiogenesis Process	Reference
HIF-1α	Regulate angiogenesis and cell invasion	[10,11,28,42,58,59]
VEGF	Inducer of angiogenesis and lymph-angiogenesis, and activates the other angiogenic stimulators	[67,68,69,70,71,72]
PDGF	Stimulates angiogenesis, regulates cell growth and division	[73,74,75,76,77]
FGF	Regulates endothelial cell proliferation, migration, and differentiation	[78,79,80,81,82]
TGF- β	Induces apoptosis, plays a tumor-suppressor role at an early stage of tumor development, and acts as a tumor promoter in the later stage of tumor progression	[83,84,85,86,87]
ANG1, ANG2	Regulates angiogenesis and stimulates matured vessel formation	[88,89,90,91,92,93,94]
EPO	Promotes erythropoiesis and tumor cell survival	[32,95,96,97,98]

Abbreviations: ANG 1: Angiopoietin 1; ANG 2: Angiopoietin 2; EPO: erythropoietin; FGF: Fibroblast growth factor; HIF-1α: Hypoxia-inducible factor-1α; PDGF: Platelet-derived growth factor; TGF: Transforming growth factor; VEGF: Vascular endothelial growth factor.

**Table 2:** The specific binding affinity of VEGF ligands and their role in the regulation of angiogenesis.

VEGF Ligands	Receptors	Role	References
VEGF-A	VEGFR-1 VEGFR-2	Proliferation of blood vessels and angiogenesis	[67,68,70,99]
VEGF-B	VEGFR-1	Proliferation of blood vessels and progression of cardiac angiogenesis	[67,99]
VEGF-C	VEGFR-2 VEGFR-3	The proliferation of blood vessels, lymph angiogenesis, and angiogenesis in the early stages of embryogenesis.	[67,99]

Abbreviations: VEGF: vascular endothelial growth factor; VEGFR: vascular endothelial growth factor receptor.

## Data Availability

Not applicable.
